# Mathematical Comparison of Time‐Based and Stroke‐Based Fatigue Models in Elite Sprint Cycling: Convergence Analysis and Practical Applications

**DOI:** 10.1111/sms.70219

**Published:** 2026-03-08

**Authors:** Anna Katharina Dunst, Vincent Scharf, Olaf Ueberschär

**Affiliations:** ^1^ Department of Endurance Sports Institute for Applied Training Science Leipzig Germany; ^2^ Department of Engineering and Industrial Design Magdeburg‐Stendal University of Applied Sciences Magdeburg Germany; ^3^ Department of Computer Science Hochschule Bonn‐Rhein‐Sieg Sankt Augustin Germany; ^4^ Directorate, Institute for Applied Training Science Leipzig Germany

**Keywords:** anaerobic performance, critical power, isokinetic sprinting, performance modeling, track cycling

## Abstract

Power decline during maximal cycling sprints is commonly modeled using either the Parallel Shift Approach (PASA), representing fatigue as a time‐dependent downward shift of the linear force‐velocity (F‐v) relationship, or the Pedal Stroke‐Based Approach (PESA), which assumes a constant relative power loss per pedal stroke (Δ). This study compared their predictive accuracy, convergence behavior, and practical applicability across sprint duration and cadences. Twelve elite track sprint cyclists (6 female and 6 male) performed 45 s maximal sprints at a fixed cadence of 135 rpm. Both models were calibrated using individual F‐v profiles and evaluated using RMSE and R^2^ . Model convergence was examined across sprint phases and cadence ranges using empirically derived and optimized parameters. Both models demonstrated excellent fit to individual sprint power profiles (R^2^ > 0.98). PASA yielded lower prediction errors (RMSE: 32 ± 11 W) than standard PESA variants (RMSE: 57–60 W, *p* < 0.001). Optimizing Δ substantially improved PESA performance (35 ± 12 W). Models converged during early sprint phases (0–15 s: 4 ± 3 W difference, *p* = 0.065) and at moderate cadences (90–130 rpm) but diverged during late phases (45 s: 22 ± 11 W, *p* = 0.013) and at high cadences (> 150 rpm). PASA and PESA provide comparable predictions under short‐duration, moderate‐cadence conditions but diverge during prolonged sprints and at extreme cadences. PASA offers higher precision for detailed fatigue analysis, whereas PESA represents a computationally efficient alternative for practice. Model selection should be guided by analytical objectives and practical constraints. Further research should prioritize validation under variable‐cadence conditions and refinement of physiological assumptions underlying sprint fatigue modeling.

## 1 Introduction

Quantifying neuromuscular fatigue during maximal sprint cycling remains a central challenge in exercise physiology, with critical implications for performance optimization, training prescription, and talent identification. Although fatigue during brief, high‐intensity efforts has been extensively studied, its mechanistic representation in mathematical models remains incomplete. Two main frameworks have been proposed to describe the progressive decline in power output: the Parallel Shift Approach (PASA), which represents fatigue as a time‐dependent downward translation of the linear force–velocity (F–v) relationship, and the Pedal Stroke‐Based Approach (PESA), which attributes fatigue to a constant relative power decrement per pedal stroke. Although often regarded as conceptually distinct, both models have demonstrated strong predictive validity, suggesting potential mathematical convergence under specific conditions.

PASA, originally validated by Buttelli et al. [1], assumes that fatigue induces a uniform downward shift of the F–v relation, preserving its linear geometry while proportionally reducing force and velocity. It has shown robust predictive performance, particularly for efforts ≥ 30 s in elite sprint cyclists and related contexts [2, 3, 4, 5, 6]. Although the force–velocity relation at the single‐muscle level is classically hyperbolic, empirical data indicate that multijoint tasks such as cycling exhibit an approximately linear F–v relationship under maximal voluntary conditions, likely due to the combined action of muscles with heterogeneous fiber‐type composition, moment arms, and coordination constraints that smooth local nonlinearities [7]. In this system‐level context, fatigue is predominantly expressed as a proportional loss of force‐generating capacity rather than a change in contractile curvature, providing a physiological rationale for PASA's assumption of a parallel downward shift and limiting the added value of hyperbolic formulations for whole‐body sprint cycling.

PESA, introduced by Tomas et al. [8], conceptualizes fatigue as the cumulative effect of maximal contractions, treating time as secondary to stroke count. Subsequent work [9, 10] reported an almost constant relative power loss per stroke across cadences, suggesting a tight coupling between cumulative neuromuscular strain and performance decline. While the underlying third‐order P‐v model remains debated, PESA's high reproducibility at the group level provides strong empirical support.

Seemingly conflicting findings have fueled debate between these perspectives. Clanet et al. [2] observed near‐parallel F‐v shifts during 60 s maximal sprints in elite track cyclists, consistent with PASA's assumptions, whereas MacIntosh et al. [11] documented non‐parallel torque‐cadence shifts, likely reflecting depletion of anaerobic work capacity (W′) and altered fiber recruitment under extreme fatigue [12], as indicated by post‐sprint optimal cadences well below those typically associated with critical power [13]. Despite these theoretical differences, both models consistently reproduce experimental data across studies and cohorts [2, 3, 4, 5, 6, 9], suggesting PASA and PESA may represent similar or, perhaps, complementary formulations of the same underlying physiological processes, distinguished primarily by their temporal and mechanical framing rather than by incompatible principles.

To date, however, no study has systematically analyzed the mathematical relationships between these models or defined the boundary conditions governing their convergence and divergence. This gap constrains both theoretical progress and practical application as practitioners lack evidence‐based criteria for model selection in different sprint contexts.

Accordingly, the present study aimed to (1) verify the mathematical equivalence of PASA and PESA under controlled conditions, (2) delineate the temporal and mechanical domains in which their predictions converge or diverge, and (3) evaluate the practical implications of model choice for sprint performance assessment. A secondary aim was to examine potential sex‐related differences in model parameters and fatigue characteristics. By addressing these aims, the study advances theoretical understanding of fatigue modeling and provides evidence‐based recommendations for model use in maximal (cycling) sprints.

## 2 Materials and Methods

### 2.1 Participants

Twelve elite track sprint cyclists (6 females: 22.2 ± 4.2 y, 171.2 ± 7.4 cm, 72.0 ± 7.2 kg; 6 males: 20.8 ± 4.2 y, 181.0 ± 8.4 cm, 84.2 ± 7.3 kg) with 4–12 years of international experience voluntarily participated in this validation study. All participants exhibited highly linear F‐v profiles (R^2^ > 0.95) and were accustomed to the testing procedures from prior laboratory assessments. Written informed consent was obtained in accordance with institutional guidelines. Procedures were approved by the ethics committee of the Institute for Applied Training Science (ER_2025.21.12_2) and conducted in accordance with the Declaration of Helsinki.

### 2.2 Experimental Design

All testing was conducted in a single session. As the protocol mirrored sprint time‐trial demands and participants were familiar with the setup from prior laboratory assessments, no additional familiarization was required. After a standardized 6‐min warm‐up at 1.0–1.5 W·kg^−1^, chosen to ensure adequate cardiovascular and neuromuscular activation without inducing fatigue, and 5 min of passive rest to allow for substantial phosphocreatine resynthesis and minimize residual metabolic fatigue, participants performed two 6 s maximal sprints against minimal resistance to obtain additional datapoints in the high‐cadence range (≥ 160 rpm), as described previously [14]. Following 5 min of active recovery at 1.0–1.5 W·kg^−1^ at self‐selected cadence and 10 min passive rest to reduce blood lactate toward baseline and restore phosphocreatine stores, participants completed a seated 45 s maximal isokinetic sprint at 135 rpm. Starting from a stationary start, they accelerated as rapidly as possible to 135 rpm at a self‐selected instant and continued maximal sprinting until task termination, while receiving strong verbal encouragement to maintain maximal power output.

All tests were performed on an FES stationary ergometer (FES, Berlin, Germany). Crank torque and angular velocity were continuously sampled at 200 Hz. Data were averaged per pedal revolution to compute cadence and mechanical power output.

### 2.3 Theoretical Framework

This study compares two deterministic models of fatigue‐induced force decline in cycling: the PASA and the PESA. Both approaches are rooted in the F‐v relationship for cycling. While Hill's hyperbolic model of muscle contraction [15] describes single‐muscle behavior, empirical evidence shows that the F‐v relationship for complex, multi‐joint tasks such as cycling is reliably linear [11, 14, 16, 17, 18, 19, 20, 21]. This linearity provides a physiologically meaningful link between mean pedal force and cadence, forming the basis of PASA [2, 3, 4, 6].

#### 2.3.1 The Parallel Shift Approach

PASA models the fatigue‐free F‐v relationship as linear:

(1) $$F\left(\mathrm{PR}\right)=a\cdot \mathrm{PR}+b$$

(2) $$P\left(\mathrm{PR}\right)=F\left(\mathrm{PR}\right)\cdot \left(2\cdot \pi \cdot r\cdot \mathrm{PR}\right)$$

where the negative slope *a* < 0 reflects the decline in mean pedal force with increasing cadence and *b* is maximal (isometric) mean pedal force (F_max_). This formulation yields key performance metrics including theoretical maximal force (F_max_ = b), theoretical maximal velocity (PR_max_ = −b·a^−1^), optimal cadence (PR_opt_ = −b·(2a)^−1^), and maximum power output (P_max_ = −b^2^·(4a)^−1^) [Equations (1), (2)].

PASA conceptualizes fatigue as a parallel downward shift of the F‐v profile over time, maintaining its geometric shape. This is supported by experimental evidence indicating proportional declines in force and velocity capabilities during repeated maximal efforts [1]. The model assumes that only the y‐intercept (F_max_) decays exponentially with fatigue, while the slope remains unchanged. Fatigue dynamics are thus described as [Equations (3), (4)]

(3a) $$F_{\max}\left(t\right)=\left(F_{\max}-C_{F}\right)\cdot e^{\min\left(0,\frac{-\left(t-\mathrm{TD}\right)}{\tau_{F}}\right)}+C_{F}$$

with limiting asymptote C_F_, time constant *τ*_F_ and a fatigue‐free delay period TD ≥ 0. The corresponding time‐dependent F‐v relation is given by:

(3b) $$F\left(t,\mathrm{PR}\right)=a\cdot \mathrm{PR}+F_{\max}\left(t\right)$$

where PR denotes pedaling rate.

#### 2.3.2 The Pedal Stroke‐Based Approach

PESA posits that fatigue is driven by the cumulative number of maximal contractions (pedal strokes), rather than elapsed time. Empirical data indicate a consistent relative power loss per stroke (Δ) across cadences, irrespective of absolute time‐dependent loss [8, 10].

In this approach, the fatigue‐free F‐v relation is modeled using Hill's rectangular hyperbola [9, 22]:

(4a) $$\left(F+c\right)\cdot \left(v+d\right)=\left(F_{\max}+c\right)\cdot d$$

Solving for force *F* gives:

(4b) $$F\left(v\right)=\frac{\left(F_{\max}+c\right)\cdot d}{v+d}-c$$

The corresponding power‐velocity (P‐v) profile is then:

(5a) $$P_{\text{peak}}\left(v\right)=F\left(v\right)\cdot v=\frac{\left(F_{\max}+c\right)\cdot d\cdot v}{v+d}-c\cdot v$$

(5b) $$\approx F_{\max}\cdot v-\frac{\left(F_{\max}+c\right)}{d}\cdot v^{2}+\frac{\left(F_{\max}+c\right)}{d^{2}}\cdot v^{3}$$

Within the relevant cadence range, this P‐v relationship can be accurately approximated by a third‐order polynomial (5b) obtained via a Taylor expansion of Equation (5a), providing an explicit analytical expression for power as a function of velocity.

In its original formulation, PESA described fatigue as a linear decline in power with increasing stroke count:

(6a) $$P\left(n,\mathrm{PR}\right)=P_{\text{peak}}\left(\mathrm{PR}\right)\;\left(1-\Delta \cdot \max\left(0,n-\mathrm{ND}\right)\right)$$

where a constant relative decrement Δ was applied per pedal stroke [10]. While this formulation captures the stroke‐dependent nature of fatigue, it inevitably leads to non‐physiological trajectories and negative power values at high stroke numbers as demonstrated in Figure B.

To ensure mathematical consistency and physiological plausibility, PESA was reformulated as an exponential decay model [Equations (6–10)]:

(6b) $$P\left(n,\mathrm{PR}\right)=P_{\text{peak}}\left(\mathrm{PR}\right)\cdot {\left(1-∆\right)}^{\max\left(0,n-\mathrm{ND}\right)}$$

where *n* is the cumulative pedal stroke count, Δ is the constant relative power loss per stroke, and ND is the stroke delay before fatigue onset. This modified exponential PESA preserves the stroke‐based scaling principle while constraining predictions to physiologically realistic behavior. All subsequent analyses therefore refer to this modified exponential formulation.

### 2.4 Model Comparison

A central link in comparing PASA and PESA is the conversion between pedal strokes (n) and elapsed time (t):

(7a) $$n\left(t\right)=\int_{0}^{t}\mathrm{PR}\left(t\right)\mathrm{dt}\approx \sum_{i=1}^{t/∆t}{\mathrm{PR}}_{i}\cdot ∆t$$

The stroke delay for fatigue onset (ND) corresponds to the number of completed pedal strokes during the temporal delay TD and is therefore given by:

(7b) $$\mathrm{ND}=n\left(\mathrm{TD}\right)$$

By substitution into (6c), PESA can be expressed in time‐based form:

(8d) $$P\left(t,\mathrm{PR}\right)=P\left(\mathrm{PR}\right)\cdot {\left(1-∆\right)}^{\max\left(0,n\left(t-\mathrm{TD}\right)\right)}$$

Conversely, PASA can be expressed per pedal stroke by converting its time constant to a stroke‐based decrement:

(9c) $$F_{\max}\left(n\right)=\left(F_{\max}-C_{F}\right)\cdot {\left(1-{∆}_{F_{\max}}\right)}^{\max\left(0,n-\mathrm{ND}\right)}+C_{F}$$

where ${∆}_{F_{\max}}=1-e^{-\frac{1}{\mathrm{PR}\cdot \tau_{F}}}$.

**FIGURE 1:**
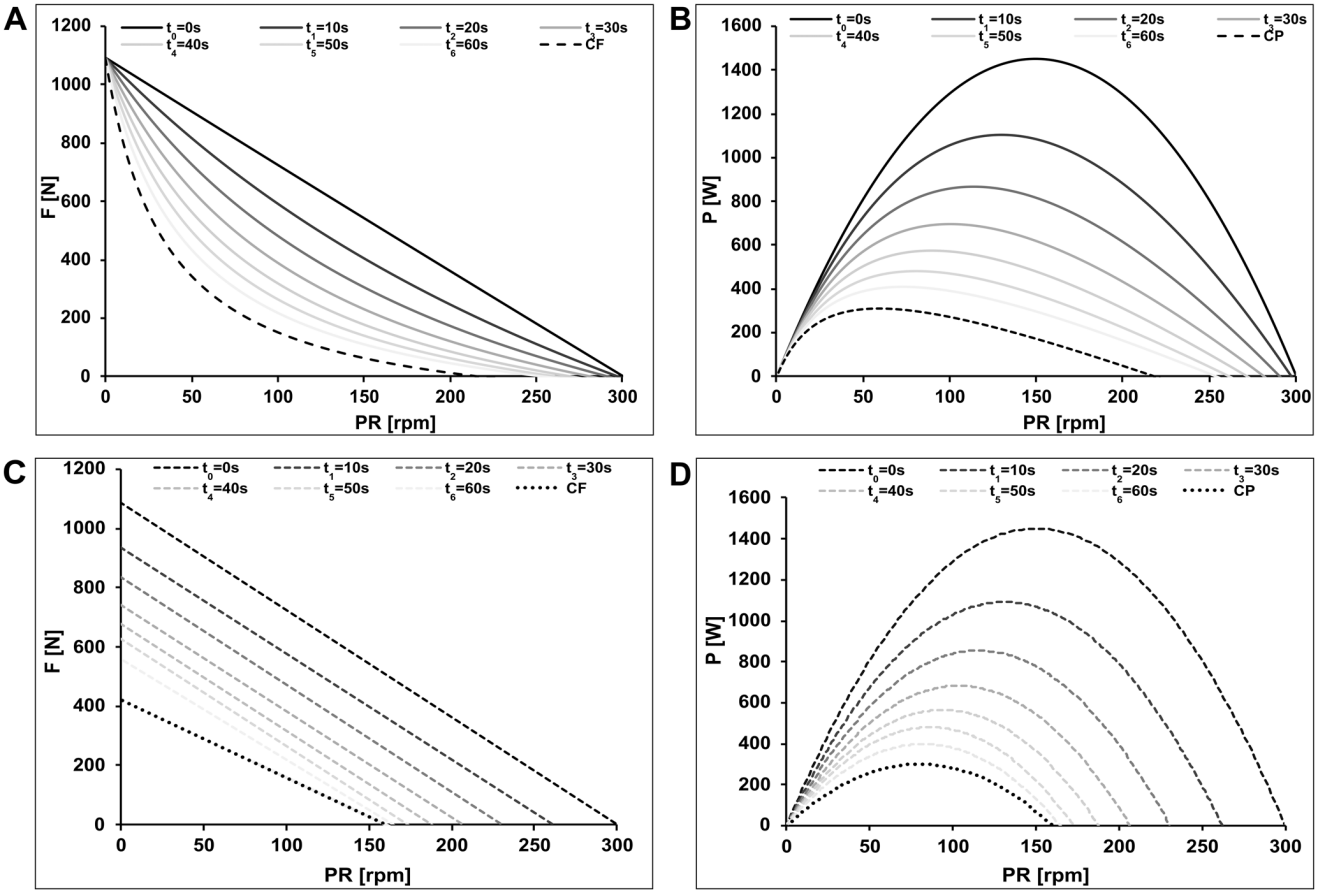
Downward shifts of the force‐velocity (F‐v; panels A, C) and power‐velocity (P‐v; panels B, D) profiles predicted by the Pedal Stroke‐Based Approach (PESA; A, B) and the Parallel Shift Approach (PASA; C, D). Both models describe an exponential time course of fatigue.

The fundamental distinction is that PASA models fatigue as a reduction of the linear F‐v profile in maximum force only (y‐intercept), while PESA models a proportional scaling of the entire hyperbolic F‐v profile. This yields different predictions for optimal cadence and mechanical efficiency under fatigue. In PASA, the reduction of the y‐axis intercept leads to a proportional reduction in both F_max_ and the theoretical maximal pedaling rate. Figure 1 illustrates the characteristic shapes and shifts in F‐v and P‐v profiles predicted by both approaches.

### 2.5 Data Processing and Statistical Analysis

Model parameters for the fatigue‐free F‐v relationship (slope a, F_max_) were estimated by combining cadence and corresponding mean pedal force from the first 3–4 pedal revolutions during acceleration (< 3 s) with 1–3 fatigue‐free pedal revolutions obtained during motoric sprints [14]. Although isolated deviations may occur during early acceleration, force–velocity parameters were determined using a best‐fit linear regression across all selected fatigue‐free data points, rendering the estimation robust to such outliers.

Model parameters for PASA (A_F_, τ_F_, C_F_, TD) were estimated using nonlinear least‐squares minimization with physiologically plausible constraints (A_F_>C_F_ > 0, 0 < τ_F_ < 100, TD ≥ 0). Model fitting minimized the sum of squared residuals between measured and modeled power output using a trust‐region reflective algorithm. Initial parameter values were derived from individual fatigue‐free force–velocity profiles and early sprint data; convergence was robust to moderate variation in initialization.

Parameters of the PESA model (Δ, ND) were derived empirically from the sprint data. The stroke delay ND was defined as the last pedal stroke preceding a sustained decline in power output, operationalized as at least five consecutive strokes with monotonically decreasing mean power. The relative decrement Δ was calculated as the mean per‐stroke power loss following ND and determined separately for the first 15 s (Δ_15_) and the full 45 s sprint (Δ_45_). To quantify PESA performance under ideal calibration conditions, an optimized PESA variant (PESAopt) was evaluated in which the decrement parameter Δ was treated as a free parameter, while the stroke delay ND was held constant.

Data processing was performed using Microsoft Excel 2016 (Microsoft Corporation, Redmond, WA, USA) and Python (Python Software Foundation), while statistical analyses were conducted in JASP (Version 0.18.2, University of Amsterdam). Normality of data distributions was assessed using the Shapiro–Wilk test, and homogeneity of variances was evaluated with Levene's test. Descriptive statistics are reported as mean ± standard deviation (SD). Statistical significance was set at α < 0.05.

Predictive accuracy of PASA and the two PESA variants (PESA_15_, PESA_45_) was evaluated using a two‐factor repeated‐measures ANOVA (within‐subject factor: Model; between‐subject factor: Sex) for both coefficient of determination (R^2^) and root mean square error (RMSE). Violations of sphericity were corrected using Greenhouse–Geisser adjustment. *Post hoc* tests employed Bonferroni‐corrected paired *t*‐tests. ANOVA effect sizes are reported as partial eta squared (η_p_^2^ = 0.01, 0.06, 0.14: small, medium, and large), and pairwise effects as Cohen's d (|d| = 0.2, 0.5, 0.8: small, medium, large).

Independent‐samples *t*‐tests (effect size: Hedges' g) were used to examine sex differences in performance metrics, model parameters, and variability of relative power loss, with Welch's correction applied for unequal variances.

The core assumption of PESA (constant relative power decrement) was tested by comparing Δ_15_ and Δ_45_ with paired *t*‐tests and variance differences with Pitman's test. Least‐squares optimization determined the decrement value (Δ_opt_) minimizing RMSE for PESA. Δ_opt_ was compared with Δ_15_ and Δ_45_ using one‐sample *t*‐tests. The optimized PESA model (PESAopt) was then compared against PASA using paired *t*‐tests.

RMSE distributions across models were illustrated using raincloud plots, integrating raw data points, boxplots, and kernel density estimates for comprehensive presentation.

## 3 Results

### 3.1 Participant Characteristics and Performance Metrics

**FIGURE 2:**
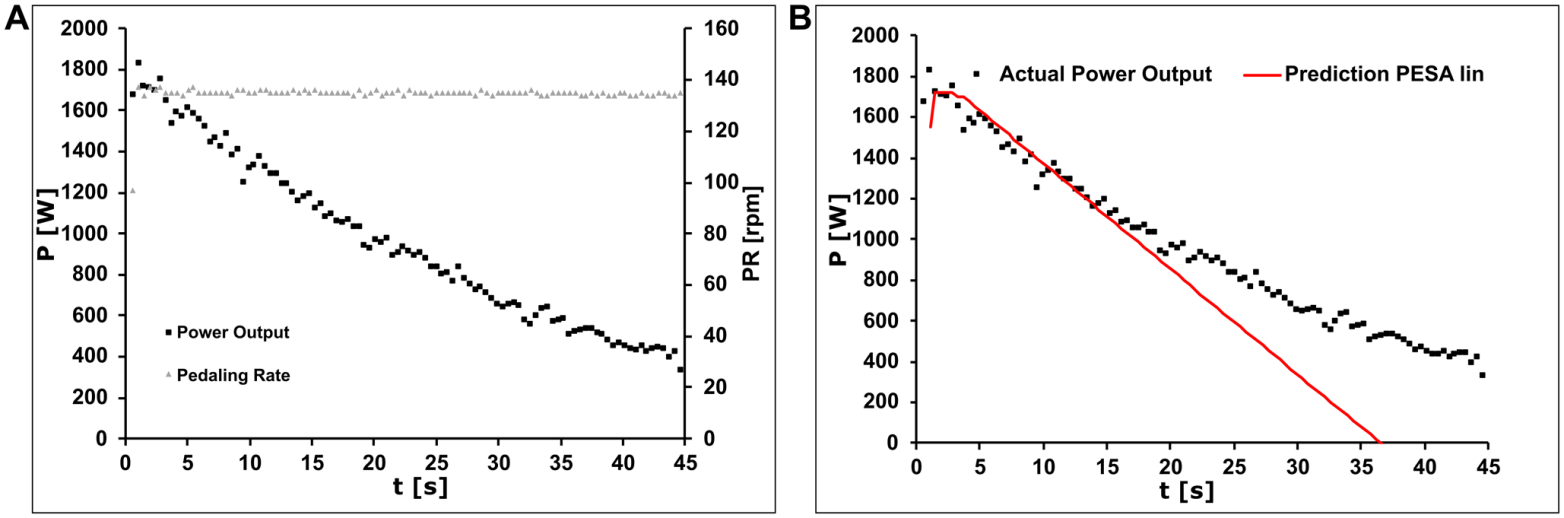
(A) Power output and cadence during a 45 s maximal sprint with a peak power of 1832 W and a mean power output of 972 W at an average cadence of 134.6 rpm. (B) Comparison between measured power output and predictions of the original linear pedal stroke–based fatigue model (linear PESA; red line). While the linear formulation approximates the initial power decline, it leads to systematic divergence and non‐physiological negative power values as sprint duration and stroke count increase, motivating the reformulation of PESA as an exponential decay model.

Twelve elite track sprint cyclists completed 45 s maximal isokinetic sprints at a fixed cadence of 134 ± 0.5 rpm. Athletes achieved a peak mean pedal force of 768 ± 183 N, a peak power output of 1395 ± 404 W, an average mean pedal force of 300 ± 82 N, and a corresponding mean power output of 709 ± 196 W. Representative time courses of power output and cadence are shown in Figure 2.

Independent samples *t*‐tests revealed clear sex‐based performance differences. Male athletes produced ~48% greater mean power than females [845 ± 178 W vs. 573 ± 89 W; *t*(10) = 3.34, *p* = 0.007, g = 1.78]. Peak power was also substantially higher in males (1648 ± 391 W vs. 1141 ± 229 W), corresponding to a 44% difference [*t*(10) = 2.75, *p* = 0.021, g = 1.46].

### 3.2 Model Calibration and Goodness‐Of‐Fit

Descriptive statistics for calibrated model parameters are presented in Table 1.

**TABLE 1:** Descriptive statistics for calibrated PASA and PESA model parameters (*n* = 12).

Category	Parameter	Mean	95% CI Mean		SD
			Lower	Upper	
F‐v	F_max_ [N]	1207	1052	1620	245
	P_max_ [W]	1443	1205	1681	375
	PR_opt_ [rpm]	133	126	140	12
	a [N rpm^−1^]	−4.53	−5.01	−4.04	0.76
PASA	A_F_ [N]	753	580	926	273
	τ_F_ [s]	38	30	46	12
	C_F_ [N]	483	401	565	129
	TD [s]	2.55	2.22	2.88	0.53
	R^2^	0.984	0.979	0.990	0.009
	P_peak_ [W]	1299	1058	1541	381
PESA	Δ_15_ [%]	1.51	1.26	1.76	0.40
	Δ_15_SD [%]	3.22	2.91	3.52	0.47
	Δ_45_ [%]	1.45	1.32	1.59	0.21
	Δ_45_SD [%]	5.74	4.85	6.63	1.40
	ND [#]	5	4	6	1

Abbreviations: a, slope of the F‐v profile; A_F_, amplitude of F_max_(t); C_F_, asymptote of F_max_(t); F_max_, maximal mean pedal force; P_max_, maximal power output; P_peak_, peak power at 135 rpm; PR_opt_, optimal pedaling rate; TD, time delay of force decay; τ_F_, time constant of F_max_(t); Δ_15_, mean relative power loss during the initial 15 s (ND‐15), with standard deviation Δ_15_SD; Δ_45_, mean relative power loss during the entire 45 s (ND‐45), with corresponding standard deviation Δ_45_SD; ND, stroke count delay of power decay.

**FIGURE 3:**
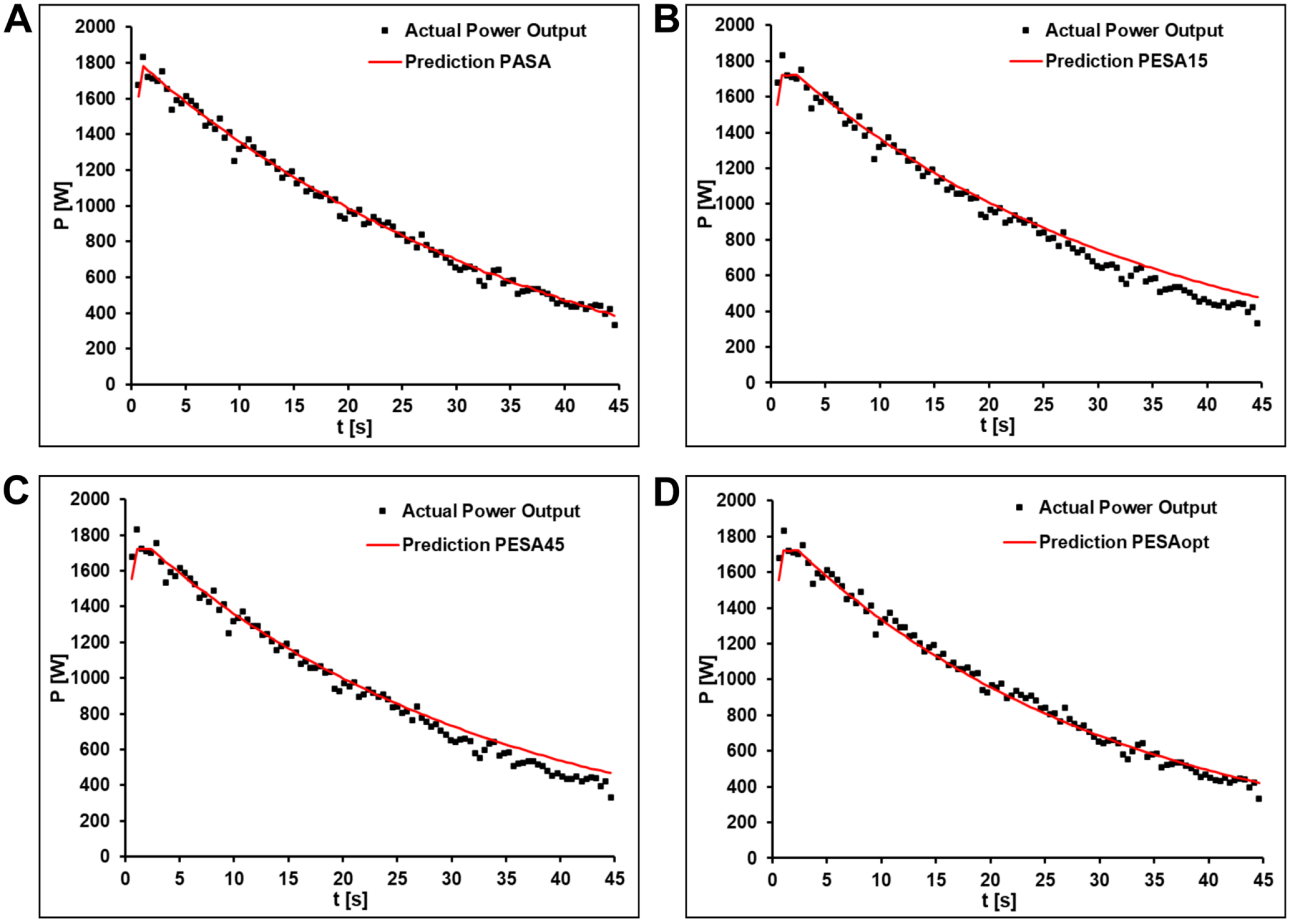
Comparison of model performance during a 45 s sprint (100 pedal strokes) using PASA, PESA15, PESA45, and optimized PESAopt. The PASA model demonstrated superior predictive accuracy (RMSE = 34 W), outperforming both standard PESA variants (RMSE = 56 W and 54 W, respectively) and the optimized PESAopt model (RMSE = 40 W).

Both PASA and PESA models demonstrated excellent fit to individual sprint power profiles, with mean R^2^ values exceeding 0.97 across all participants. A repeated‐measures ANOVA revealed no significant differences in goodness‐of‐fit between model configurations (Mean R^2^ = 0.986 ± 0.006; F = 1.282, *p* = 0.299, η_p_^2^ = 0.114), indicating that all models adequately captured the underlying power–time relationship during sprint exercise. A representative comparison of model fits is presented in Figure 3.

### 3.3 Predictive Accuracy Comparison

Despite equivalent model fit, predictive accuracy differed significantly between approaches [F(2, 20) = 25.78, *p* < 0.001, η_p_^2^ = 0.701]. PASA yielded the lowest prediction error (RMSE = 32.0 ± 10.6 W), while both PESA variants demonstrated significantly higher errors (PESA15: 56.6 ± 15.8 W; PESA45: 59.8 ± 14.4 W; both *p* < 0.001, Cohen's d ≥ 1.79). No difference was observed between PESA calibration approaches (*p* = 0.560, d = 0.20). Model performance hierarchy remained consistent across sex (model × sex interaction: *p* = 0.117, η_p_^2^ = 0.201).

**FIGURE 4:**
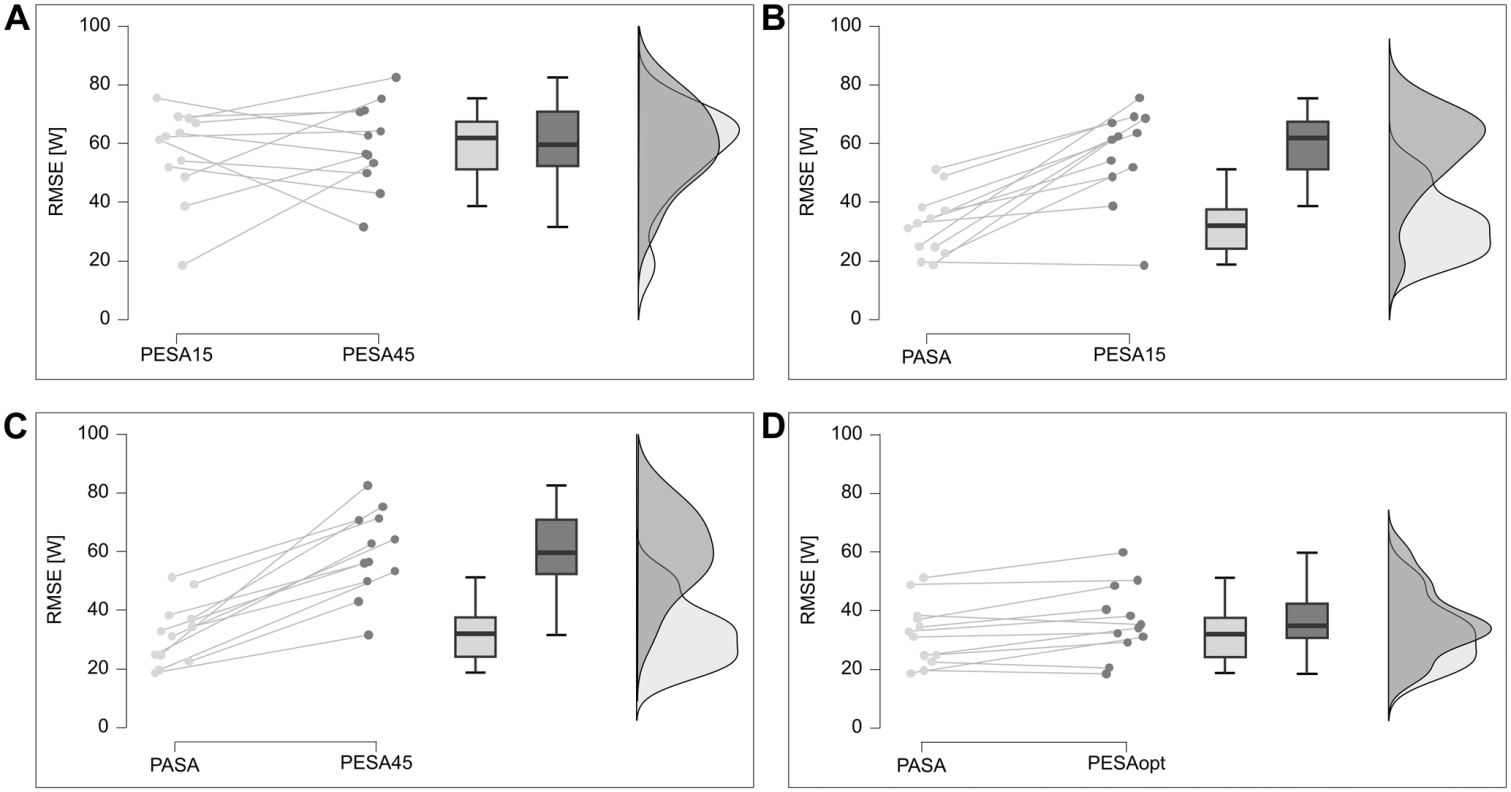
Distribution of prediction errors (RMSE) for PESA15, PESA45, PESAopt, and PASA. Raincloud plots display individual data points, boxplots, and kernel density estimates to illustrate central tendency, variability, and distributional characteristics.

Optimization of the PESA decrement parameter (Δ_opt_ = 1.57 ± 0.16%) substantially improved its predictive accuracy, reducing the RMSE to 35 ± 12 W (PESAₒₚₜ). This optimized model performed significantly better than empirical PESA variants (*p* < 0.001, d ≥ 1.30) and achieved predictive accuracy comparable to that of PASA, with no statistically significant difference between them (mean RMSE difference: 5 ± 3 W; *p* = 0.056, d = −0.618). The mean absolute difference in instantaneous power output prediction between the optimized PESA and PASA models was 2 ± 8 W. Figure 4 presents the distribution of RMSE across all models.

### 3.4 Model Convergence and Divergence

The practical convergence zone between PASA and PESA is defined as the range of cadence–time combinations in which differences between model predictions remain below the measurement precision. Across all experimental conditions, both models showed strong overall agreement, but systematic deviations emerged as a function of cadence and sprint duration.

#### 3.4.1 Convergence and Divergence Across Pedaling Rates

**FIGURE 5:**
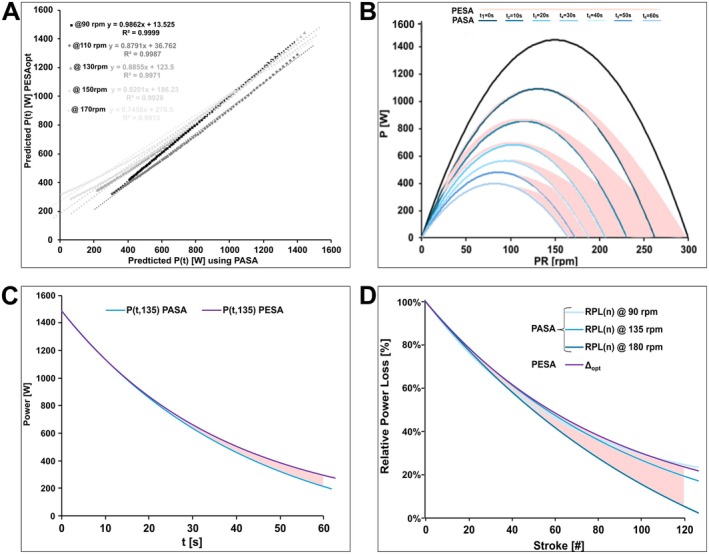
Model convergence and divergence of PASA and PESA across cadence and time. (A) Linear regression between PASA and PESA power predictions at four cadences shows near‐perfect agreement at moderate cadence but increasing divergence at higher cadences. (B) Differences in 10 s segments over a 60 s sprint on the power‐velocity‐time surface. (C) Power output prediction at 135 rpm highlights initial similarity, with divergence after ~15–20 s. (D) Comparison of stroke‐based relative power loss reveals PASA's cadence‐dependent loss rates (90, 135, 180 rpm; colored lines) versus PESA's constant per‐stroke decrement (Δ_opt_). The shaded region marks the divergence zone, emphasizing increased discrepancy at higher cadences due to greater stroke accumulation per unit time.

Linear regression analyses revealed near‐perfect functional equivalence at moderate pedaling rates (90 rpm; slope = 0.99, intercept = 13.5 W, R^2^ = 0.999) as model predictions closely followed the identity line. However, as cadence increased (110–170 rpm), the models progressively diverged: regression slopes decreased to 0.89–0.75 and intercepts rose to 37–217 W (R^2^ = 0.991–0.999; Figure 5A). These findings indicate that PESA consistently predicts higher power outputs than PASA as cadence rises (Figure 5B).

#### 3.4.2 Convergence and Divergence Across Sprint Phases

During the early sprint phase (0–15 s), absolute differences between models were small (4.0 ± 2.8 W; *p* = 0.065, d = 0.59), and convergence persisted through mid‐sprint (30 s: 2.4 ± 1.9 W; *p* = 0.151, d = 0.45). This initial similarity reflects the quasi‐linear portion of the exponential decay and the near‐quadratic shape of PESA's P‐v curve, rendering the two models effectively equivalent at sprint onset. Substantial divergence appeared only toward sprint termination (45 s: 22 ± 11 W; *p* = 0.013, d = 0.86), reflecting the models' asymptotic behavior: PASA approaches a finite residual force (C_F_), whereas PESA decays multiplicatively toward zero without a physiological floor (Figure 5C).

Together, these results demonstrate that model agreement is high at moderate cadences and early sprint phases but declines systematically with increasing cadence and exercise duration, with PESA tending to yield higher power predictions under these conditions (Figure 5B).

### 3.5 Sex‐Related Differences in Model Parameters

Baseline neuromuscular characteristics differed markedly between sexes. Males demonstrated higher maximal power (1729 ± 234 vs. 1157 ± 240 W; *p* = 0.002, g = 2.23), maximal mean pedal force (1389 ± 157 vs. 1026 ± 164 N; *p* = 0.003, g = 2.09), optimal pedaling rate (140 ± 10 vs. 126 ± 10 rpm; *p* = 0.034, g = 1.31), and steeper F‐v relationships (−4.98 ± 0.63 vs. −4.07 ± 0.62 N·rpm^−1^; *p* = 0.030, g = 1.35).

Fatigue characteristics were largely sex‐invariant across both modeling approaches. PASA parameters showed no significant sex differences in time constants (τ_F_: 39.1 ± 9.2 vs. 36.5 ± 15.5 s; *p* = 0.732, g = 0.19) or curvature factors (C_F_: 495 ± 151 vs. 470 ± 117 W; *p* = 0.755, g = 0.17), with only amplitudes differing significantly (A_F_: 938 ± 237 vs. 567 ± 158 W; *p* = 0.010, g = 1.70). Similarly, PESA decrement rates were equivalent between sexes (Δ15: 1.45 ± 0.31 vs. 1.57% ± 0.50%, *p* = 0.611; Δ45: 1.44 ± 0.18 vs. 1.46% ± 0.26%, *p* = 0.886), as was associated variability (*p* = 0.843).

Both models demonstrated equivalent predictive accuracy across sexes (PASA: males 31.2 ± 12.1 vs. females 32.8 ± 9.4 W, *p* = 0.784; PESA15: males 58.1 ± 18.2 vs. females 55.1 ± 13.8 W, *p* = 0.723), indicating universal applicability regardless of sex‐specific baseline performance differences.

## 4 Discussion

This study presents the first comprehensive mathematical and experimental comparison of two principal models for neuromuscular fatigue during maximal sprint cycling: the Parallel Shift Approach (PASA) and the Pedal Stroke‐Based Approach (PESA). A key finding is that these ostensibly incompatible frameworks demonstrate statistical equivalence under specific conditions, with both achieving excellent explanatory power (R^2^ > 0.98) and high predictive accuracy (RMSE ≤ 5%) under isokinetic conditions, despite divergent conceptual foundations. Although PASA showed superior predictive accuracy in its standard form, mathematical optimization of PESA's per‐stroke decrement parameter (Δₒₚₜ) eliminated this advantage, indicating that model selection should be guided by practical application needs rather than presumed theoretical superiority.

### 4.1 Practical Equivalence and Theoretical Reconciliation

Convergence–divergence analysis revealed a close agreement between PASA and PESA predictions at moderate cadences (90–130 rpm) and during early sprint phases (0–30 s), with systematic deviations emerging at higher cadences and longer durations (Figure 5). Initial functional equivalence arises from PASA's near‐constant relative force loss and PESA's quasi‐quadratic P‐v profile at sprint onset. Beyond approximately 30 s, however, their predictions diverge due to fundamentally distinct asymptotic behaviors and physiological assumptions.

PASA's decay toward a finite residual force (C_F_) implies preserved oxidative motor‐unit recruitment as glycolytic fibers fatigue, aligning with Henneman's size principle and the critical‐power framework [23, 24]. In contrast, PESA's multiplicative decline toward zero lacks a physiological lower bound, potentially overestimating fatigue during sustained efforts where aerobic metabolism dominates force maintenance. Given strong empirical support for the critical‐power concept, incorporating a critical‐force asymptote into both models would improve physiological realism. Such an integration would explicitly link performance decline to anaerobic work capacity depletion while accounting for sustainable aerobic power, thereby reconciling both approaches within a unified bioenergetic framework.

### 4.2 Model Convergence and Divergence and Underlying Physiology

Mathematical analysis confirmed that PASA and PESA are mathematically equivalent only at specific cadence‐time combinations:

(10a) $${\left(1-∆\right)}^{n\left(t\right)}=\frac{a\cdot \mathrm{PR}+F_{\max}\left(t\right)}{a\cdot \mathrm{PR}+F_{\max}}$$

with congruence at:

(10b) $$\tau \approx \frac{1}{\mathrm{PR}\cdot \ln\left(\frac{1}{1-∆}\right)}$$

This congruence occurs at a single cadence per parameter set, providing a direct mathematical explanation for the systematic divergence observed experimentally (Figure 5D). Outside these precise conditions, relative prediction errors (ϵ < 0.05) define practical corridors where model deviations remain below 5%, representing regions of reliable interchangeability. Beyond this error boundary, particularly at very high cadences, the predictions of the two models diverge substantially. Empirical comparisons reveal substantial overlap between PASA and PESA within the cadence and duration ranges most relevant to competitive sprint cycling. In these intervals, PASA's cadence‐time trajectory closely matches PESA's assumption of a constant relative power loss per pedal stroke (Figure 5B), a finding reflected in high agreement and predictive accuracy across typical performance contexts. However, outside this corridor, especially at elevated cadences, model differences become pronounced. These discrepancies are rooted in the models' divergent mathematical bases: PASA's parallel shift of a linear F‐v profile versus PESA's stroke‐invariant relative power decrement applied to a third‐order polynomial P‐v profile (Figure 5B). Since the true physiological mechanism remains unknown, this divergence does not necessarily invalidate either model but rather highlights the distinct and competing hypotheses they represent regarding the nature of neuromuscular fatigue.

Current evidence does not unequivocally favor either model's physiological accuracy. Recent data from track cycling suggest that fatigue induces a near‐parallel shift in the linear F‐v profile during a 1000 m time‐trial [2]. PASA's predicted parallel shift aligns with established muscle physiology, whereby fatigue preferentially impairs high‐threshold glycolytic fibers while preserving the intrinsic contractile properties of active motor units [25, 26]. This selective impairment maintains the geometry of the F‐v relationship while proportionally reducing both force and velocity, a mechanism supported by single‐fiber studies [12]. The linearity of the F‐v profile in multi‐joint movements can be physiologically explained by overlapping cross‐bridge attachment phases that compensate for single‐fiber detachment phases during muscle work [7], and was also observed under fatigue [11].

PESA's central assumption of cadence‐independent, constant power decrement per pedal stroke received mixed empirical support. While Δ stabilized at approximately 1.5%–1.6%, stroke‐to‐stroke variability increased substantially with fatigue progression, compromising predictive reliability. This variability explains PESA's systematic underestimation of late‐sprint fatigue despite apparent validity at the group mean level.

**FIGURE 6:**
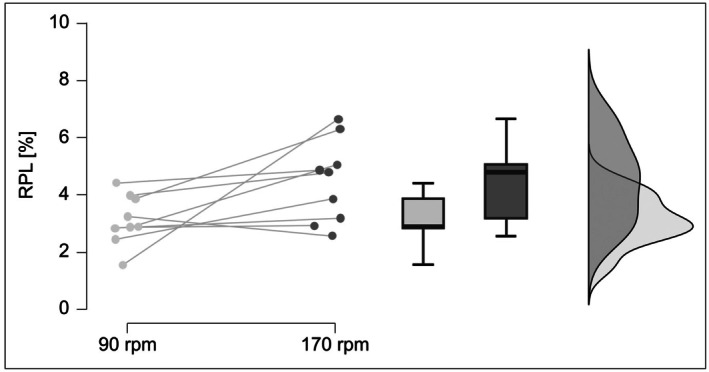
Comparison of relative power loss during isokinetic maximal cycling sprints performed at 90 rpm and 170 rpm. Data originate from Haase et al. [27] and were re‐analyzed for the present study.

Secondary analysis of Haase et al. [27] data revealed systematic differences in relative power loss (RPL) between 90 rpm and 170 rpm during 10 s maximal isokinetic cycling sprints Figure 6. Higher decrement rates at 170 rpm directly challenge PESA's assumption of cadence‐independent per‐stroke decline, suggesting the model's applicability may be limited to specific cadence ranges.

Conversely, theoretical modeling in endurance contexts indicates that a third‐order polynomial may enhance model fit [28]. PESA's stroke‐counting mechanism, while less intuitively aligned with classical fatigue theory, may reflect the cumulative metabolic cost of repeated maximal contractions. The consistent relative power loss per stroke across cadences implies a mechanistic link between neuromuscular strain and performance decline that transcends time alone, a concept supported by molecular exercise physiology, where the accumulation of metabolic byproducts that cause contractile dysfunction is more closely linked to the total work performed than to time itself [25, 26].

The increasing discrepancy under extreme conditions emphasizes the need for further empirical research to evaluate which set of assumptions, if any, more accurately reflects the underlying biology of fatigue during maximal cycling efforts. The high yet incomplete variance explained by both models (*R* ^2^ < 1) indicates that neither fully captures true fatigue dynamics; both remain approximations. Therefore, future research should also pursue hybrid modeling strategies to improve predictive accuracy across broader cadence and duration ranges.

### 4.3 Sex Differences in Fatigue Kinetics

Consistent with previous reports, males displayed higher absolute maximal force, power, and optimal cadence [28, 29]. However, fatigue kinetics themselves, captured by both decrement and decay constants, were remarkably similar across sexes, which is in line with previous findings [29]. These findings support the interpretation that sex differences in sprint cycling are attributable primarily to baseline power‐generating capacity rather than differential fatigue resistance under maximal conditions.

### 4.4 Practical Applications

Our convergence–divergence analysis provides clear guidance for model selection across different performance contexts. For short‐to‐moderate duration assessments (≤ 30 s) at moderate cadences (90–130 rpm), both models yield functionally equivalent predictions, allowing practitioners to choose based on practical considerations. PASA offers superior predictive accuracy in standard implementations and physiologically meaningful asymptotic behavior, while PESA provides conceptual simplicity and direct quantification of neuromuscular strain accumulation.

For prolonged sprints (> 30 s) or high‐cadence efforts (> 150 rpm), PASA likely demonstrates superior predictive accuracy and greater physiological plausibility. Under these conditions, PESA probably systematically overestimates fatigue accumulation, potentially leading to substantial errors in performance prediction and training prescription. Thus, PASA represents the preferred modeling framework for analyzing extended or high‐cadence maximal efforts.

In contrast, PESA remains particularly valuable for rapid diagnostic applications. The 15 s assessment protocol enables frequent monitoring while minimizing athlete fatigue during testing sessions. Furthermore, PESA provides directly interpretable per‐stroke decrement values without requiring complex parameter optimization, though this simplicity comes at the cost of mechanistic depth. When critical power is known, PESA also facilitates straightforward estimation of anaerobic work capacity through intuitive per‐stroke decrement calculations, bypassing the need for sophisticated exponential fitting procedures.

The temporal specificity of model performance has important practical implications across sporting disciplines. Track cycling sprint events (typically 17–60 s) particularly benefit from PASA's accuracy during extended efforts. However, numerous other sports featuring brief sprints (< 30 s), including team sports, racket sports, and explosive field events, can leverage PESA's practical advantages without sacrificing predictive accuracy. This distinction enables practitioners to align model selection with both the physiological demands of the sport and the practical constraints of the testing environment.

The substantial improvement in PESA's predictive accuracy following parameter optimization highlights the critical importance of robust calibration protocols. Although empirically derived decrement rates aligned with optimized values, the significantly increased variability observed during prolonged efforts reflects growing inter‐individual heterogeneity in fatigue trajectories. This finding suggests that individualized calibration procedures may be essential for maximizing predictive accuracy in applied contexts, particularly when assessing fatigue dynamics across diverse athletic populations.

### 4.5 Limitations and Future Directions

This study was conducted under highly controlled isokinetic conditions in national elite‐level track sprint cyclists, which limits generalizability to variable‐cadence efforts, field environments, and sub‐elite or recreational populations. Although sex‐based analyses were included, the small subgroups (*n* = 6) reduce power to detect subtle differences in fatigue kinetics and necessitate cautious interpretation.

Within the tested conditions, PASA and PESA showed strong overall agreement, indicating that both frameworks capture key features of fatigue development during maximal sprint cycling. Systematic divergence at longer sprint durations and extreme cadences, however, reflects fundamental differences in model structure and fatigue representation rather than measurement noise or fitting instability. Consequently, both approaches should be regarded as phenomenological models that describe observable fatigue patterns, not as fully mechanistic representations of underlying physiology.

Extrapolation beyond the experimentally tested cadence of 135 rpm represents a further limitation. Predictions at markedly lower or higher cadences rely on the assumption of cadence‐invariant fatigue parameters in both PASA and PESA. If fatigue kinetics are cadence dependent in vivo, especially at very high cadences, true convergence boundaries may differ from those estimated here, constraining the interpretability of model behavior under less common conditions without explaining the structural divergence itself.

Parameter identifiability also limits practical applicability. The optimized decrement Δ_opt_ is a theoretical upper bound of PESA performance under ideal calibration, not a directly deployable diagnostic metric. At present, neither the stroke‐based decrement Δ in PESA nor the time constant τ_F_ in PASA can be robustly specified a priori without observing a substantial portion of a maximal sprint, reflecting an inherent trade‐off between test duration and prediction accuracy. Future work should determine the minimum sprint duration required to obtain stable fatigue parameters suitable for reliable extrapolation across the power–duration spectrum.

Future research should therefore prioritize systematic validation of fatigue models across the full power–velocity–duration space, with particular emphasis on very low and very high cadences, where model assumptions diverge most and empirical data remain sparse. Advancing fatigue modeling will further require tighter integration with muscle‐fiber‐level and metabolic measurements. Incorporating direct metabolic markers into PASA may increase its physiological specificity, while deeper analysis of PESA's stroke‐based formulation could clarify how cumulative neuromuscular strain and metabolite accumulation drive performance decline. Ultimately, hybrid approaches that combine PASA's mechanistic rigor with PESA's stroke‐based pragmatism appear most promising for improving predictive accuracy across cadences, sprint durations, and athlete populations.

## 5 Conclusion

PASA and PESA are both valid, physiologically grounded models of fatigue in sprint cycling when appropriately calibrated. PASA provides slightly higher predictive accuracy under extreme conditions, whereas PESA offers practical advantages through shorter testing duration, direct estimation of W′, and ease of implementation, making it well suited for routine performance monitoring. Their strong convergence across sport‐relevant cadences and sprint durations indicates that both models capture key aspects of fatigue development and should be regarded as complementary rather than competing frameworks, with model choice guided by application context.

Nevertheless, the physiological mechanisms underlying fatigue and their biomechanical representation are not yet fully understood. Further work is required to refine model assumptions and evaluate their validity across the full power–velocity–duration spectrum. In particular, experimental validation under variable‐cadence conditions and at very low and very high pedaling rates should be prioritized as these conditions expose the greatest model discrepancies and remain empirically underexplored.

## 6 Perspective

This study provides the first systematic comparison of two deterministic fatigue models in sprint cycling, demonstrating their practical equivalence across cadences and durations typical of elite performance. These findings contribute to ongoing efforts in exercise physiology to establish valid and efficient methods for quantifying neuromuscular fatigue and short‐term power decline [4, 8, 10].

The convergence boundaries between PASA and PESA highlight unresolved questions regarding how fatigue alters the force–velocity relationship in multi‐joint exercise. Muscle‐level evidence indicates that fatigue reduces maximal force, slows shortening velocity, and alters cross‐bridge kinetics [30], aligning conceptually with PESA's proportional scaling. However, the well‐established linear force–velocity profile in cycling and its parallel downward shift with fatigue provide strong empirical support for PASA as a robust system‐level descriptor. Targeted studies using maximal efforts at extreme movement velocities are needed to clarify the underlying mechanisms of fatigue in multi‐joint tasks.

Integrating PASA and PESA within the critical power framework could extend traditional power–duration modeling toward a three‐dimensional power–velocity–duration representation. Such integration may also enable more efficient estimation of CP and W' from short sprint protocols, enhancing both the accuracy and practicality of athlete profiling.

## Author Contributions

A.K.D. designed and conducted the study. A.K.D. and V.S. performed data analysis. A.K.D. and V.S. developed the modeling framework and drafted the manuscript. O.U. supervised the project, contributed to model development, and critically revised the manuscript for important intellectual content. All authors (A.K.D., V.S., and O.U.) reviewed and approved the final manuscript for publication.

## Funding

This research was funded by the Federal Ministry of the Interior, Germany (Bundesministerium des Innern, für Bau und Heimat) through project AD‐5‐25.

## Disclosure

*Code Availability*: The Python scripts used for data processing and model analysis, together with an example dataset, will be made publicly available online to enhance reproducibility.

## Ethics Statement

All procedures were approved by the institute's ethics committee (Institute for Applied Training Science, Leipzig, Germany; ER_2025.21.12_2) and conducted in accordance with the Declaration of Helsinki.

## Consent

All participants provided written informed consent prior to participation.

## Conflicts of Interest

The authors declare no conflicts of interest.

## Data Availability

The data that support the findings of this study are available on request from the corresponding author. The data are not publicly available due to privacy or ethical restrictions.
